# Case Report: Surgical Treatment of Type IV Spinal Dermoid Sinus in a Shiba Inu

**DOI:** 10.3389/fvets.2022.849025

**Published:** 2022-03-23

**Authors:** Kaho Takahashi, Shintaro Kimura, James K. Chambers, Yukiko Nakano, Takeshi Ishikawa, Sadatoshi Maeda, Hiroaki Kamishina

**Affiliations:** ^1^Joint Department of Veterinary Medicine, Faculty of Applied Biological Sciences, Gifu University, Gifu, Japan; ^2^The United Graduate School of Veterinary Sciences, Gifu University, Gifu, Japan; ^3^The Animal Medical Center of Gifu University, Gifu University, Gifu, Japan; ^4^Laboratory of Veterinary Pathology, Graduate School of Agricultural and Life Sciences, The University of Tokyo, Tokyo, Japan; ^5^Aichi Dog and Cat Medical Center, Aichi, Japan

**Keywords:** dermoid sinus, dog, surgical treatment, vertebral malformation, meningomyelitis

## Abstract

A 2-year-old spayed female Shiba Inu was presented with progressive non-ambulatory bilateral paraparesis, back pain, and urinary incontinence. CT and MRI revealed multiple vertebral malformations and type IV dermoid sinus. Hemilaminectomy was performed in T1–T5 to remove the dermoid sinus and granulomatous lesion that infiltrated into the spinal cord parenchyma. Histopathological examination of the excised tissue revealed type IV dermoid sinus with granulomatous meningomyelitis. After surgery, back pain was resolved, and the dog recovered ambulation and voluntary urination at the time of follow-up 4 months after surgery.

## Introduction

Dermoid sinus is a congenital malformation resulting from failure of separation of the neural tube from the skin ectoderm during embryonic development (1, 2). A previous study of the Rhodesian ridgeback population reported an overall prevalence of dermoid sinus of 5.3% and that it is considered hereditary (3). In Rhodesian ridgeback, the dorsal hair ridge was reported to be caused by a dominant 133-kb duplication involving three fibroblast growth factor (FGF) genes and it was proposed that its duplication was associated with the development of dermoid sinus (4). However, a recent study concluded that the 133-kb duplication that forms the dorsal hair ridge is not identical with the hypothesized locus for dermoid sinus (5). Dermoid sinus has been reported in several other breeds; however, there are no reports in Shiba Inu. Six different types of dermoid sinuses have been described depending on the extent of penetration into the subcutaneous tissues (6–9); Type I lesions extend to the supraspinous ligament; type II lesions do not extend as far as the supraspinous ligament, but are connected to it by a fibrous band; type III lesions do not connect to the supraspinous ligament; type IV lesions attach to the dura mater; type V lesions have no connection to the skin surface; type VI lesions have the open sinus tracts reaching the level of the supraspinous ligament and continuous to the dura mater by a fibrous band. Presumptive diagnosis is based on clinical findings and results of CT and MRI, and definitive diagnosis is based on histopathological examination of surgically resected tissue (10). In type IV or VI dermoid sinus, meningitis or meningomyelitis secondary to bacterial infection may occur due to the communication between the sinus and the dura mater, resulting in neurological signs (9, 11). The surgical treatment is a preferred option in case of dermoid sinuses with neurological deficits (12).

This case report describes the clinical presentation, imaging findings, surgical treatment, and outcome of type IV dermoid sinus with severe granuloma secondary to bacterial infection and spinal malformations in a Shiba Inu.

## Case Description

**Figure 1:**
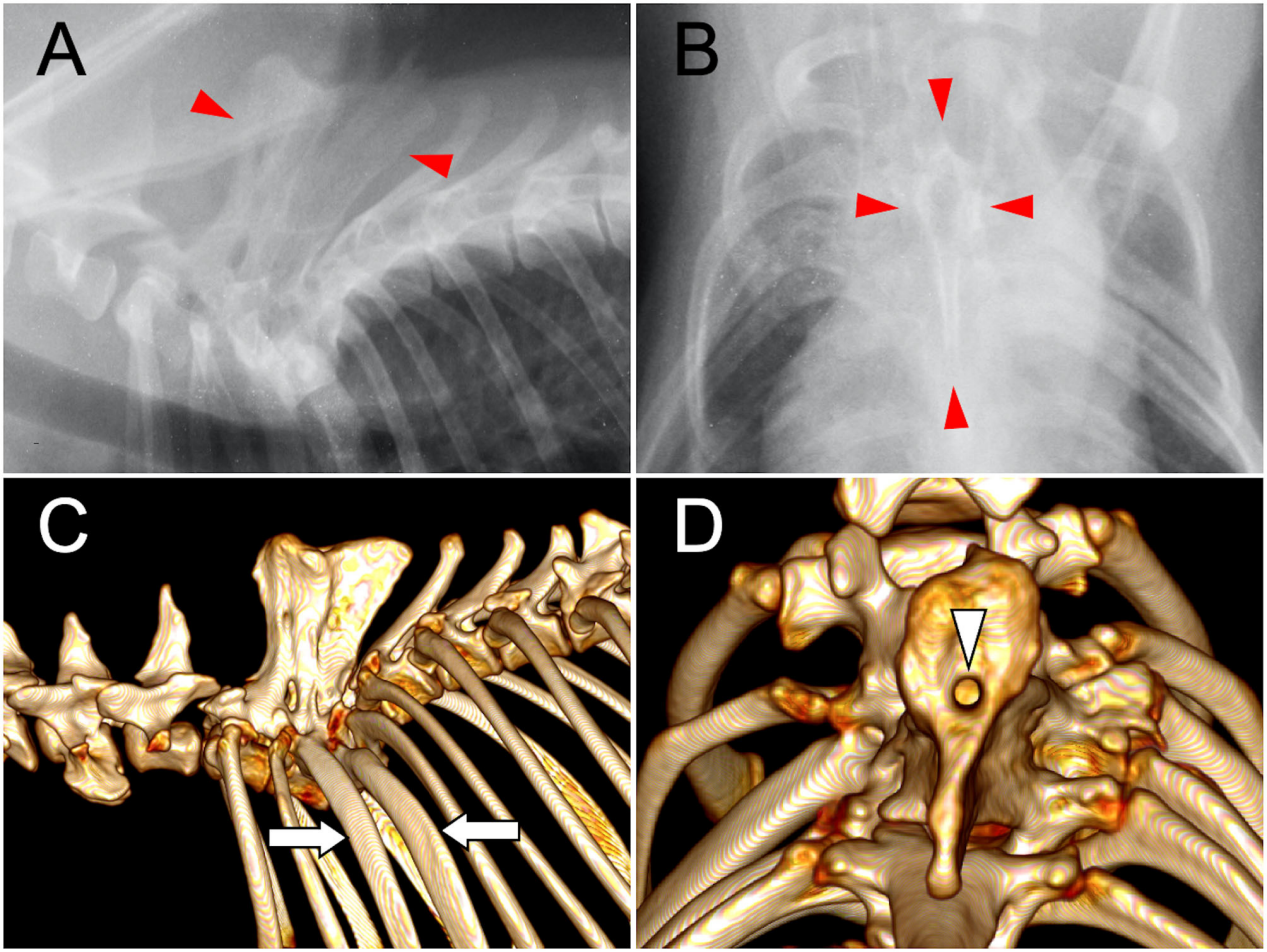
Plain radiographs and CT images of the thoracic spine at the referral hospital. Right lateral **(A)** and ventrodorsal **(B)** radiographs showed severe lordosis and conjoined spinous process of T1–T5 (arrowheads). Reconstructed three-dimensional CT images of the thoracic vertebrae **(C,D)**. The spinous processes of T1–T5 were fused and the ribs of T3 and T4 were abnormally thickened (arrows) **(C)**. On the dorsal view, the tubular opening (arrow heads) of the conjoined spinous process is seen **(D)**.

A 2-year and 3-month-old spayed female Shiba Inu was presented to a local veterinary hospital in Toyota city with back pain and shivering. The dog was referred to the Aichi Dog and Cat Medical Center at the age of 2 years and 8 months. No abnormality was noted on neurological examination except for back pain. Plain radiography and CT revealed severe lordosis of the thoracic vertebrae and fused spinous processes between T1 and T5. There were only 11 thoracic vertebrae, and the left T3 and T4 ribs were abnormally thickened (Figure 1).

On MRI, sagittal and transverse T2-weighted, T1-weighted, and T1-weighted post-contrast imaging were acquired. The spinal cord at the level of the T5–T6 vertebrae was hypointense on sagittal T2-weighted imaging and isointense on sagittal T1-weighted imaging. This region was enhanced with contrast medium (0.2 ml/kg, ProHance, Bracco-Eisai, Tokyo, Japan) intravenous administration. An epidural fat tissue exhibited as a hyperintense triangular structure on T2-weighted imaging was observed ventral to the focally enlarged spinal cord at the level of the lesion. Cisternal cerebrospinal fluid (CSF) analysis showed elevated total cell count of 58 cells/μL (reference range 0–5 cells/μL) with neutrophilic pleocytosis (~50%). Bacterial culture of CSF was negative. Orbifloxacin (5 mg/kg q24, VICTAS SS Tablets, DS Pharma Animal Health, Osaka, Japan), Pregabalin (4.6 mg/kg q12, Lyrica, Viatris, Tokyo, Japan), and Firocoxib (7.0 mg/kg, q24, Previcox, Nippon Zenyaku Kogyo, Fukushima, Japan) were prescribed. After 6 days, back pain did not improve, and progressed to hindlimb paresis and urinary incontinence.

At the age of 2 years and 9 months, the dog was referred to the Animal Medical Center of Gifu University for further evaluation. At the time of presentation, the dog exhibited non-ambulatory paraparesis and back pain. Other physical examination findings were unremarkable. Neurological examination revealed normal mental status and the results of cranial nerve examination were normal. The postural reactions of both hindlimbs were absent and the patellar tendon reflexes were increased. Based on the neurological examination, location of the lesion was suspected to be the T3-L3 spinal cord segment and differential diagnosis included congenital, inflammatory, and degenerative diseases. Complete blood count (CBC) and blood biochemical examination were unremarkable.

**Figure 2:**
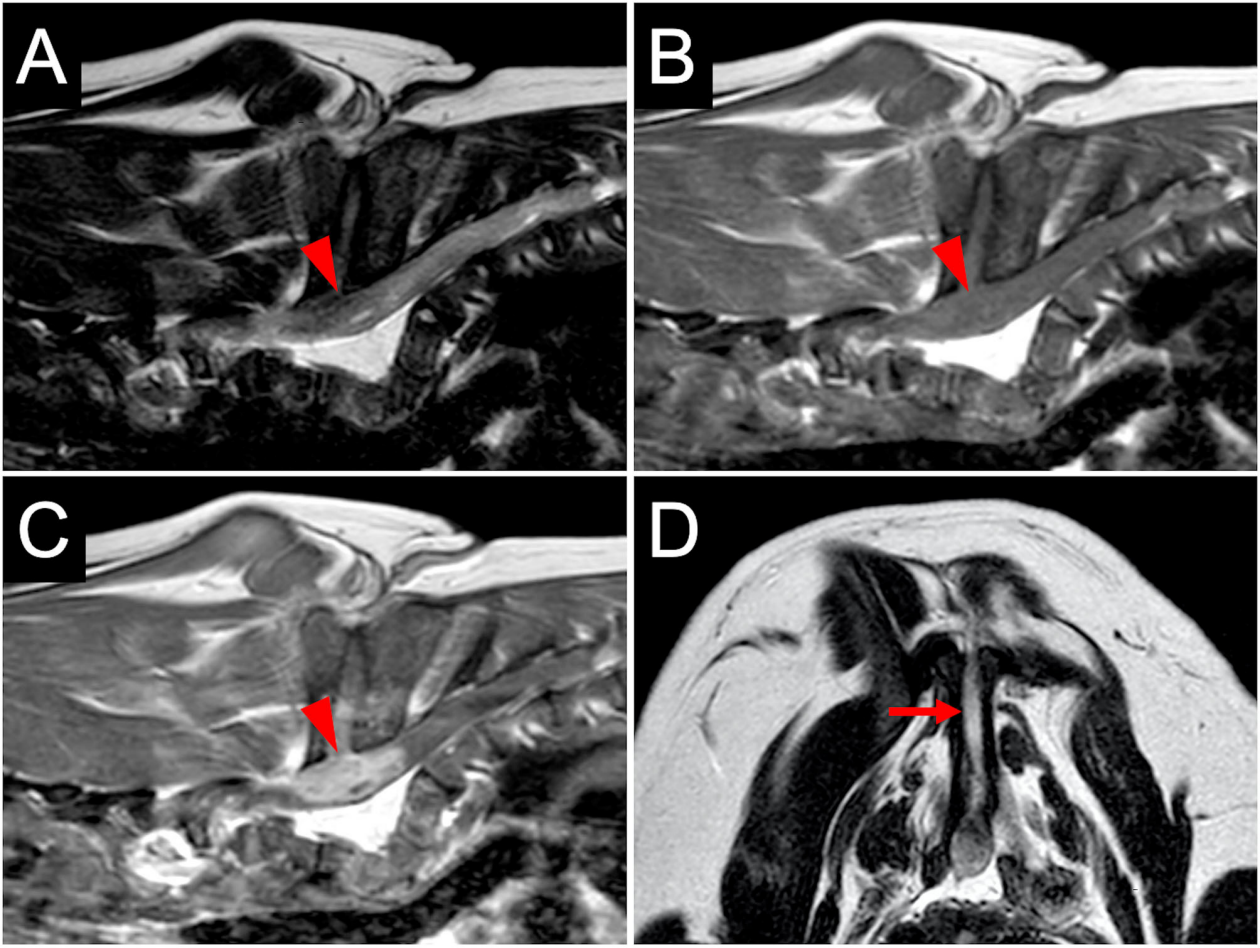
MR images at diagnosis. The spinal cord at the level of T5–T6 vertebrae (arrowheads) is hypointense on T2-weighted sagittal imaging **(A)** and isointense on T1-weighted sagittal imaging **(B)**. T1-weighted post-contrast sagittal imaging **(C)** shows the enhanced lesion in the spinal cord with contrast medium. Transverse T2-weighted imaging at the level of T5–T6 **(D)** shows a sinus tract (arrow) in the fused spinous processes.

**Figure 3:**
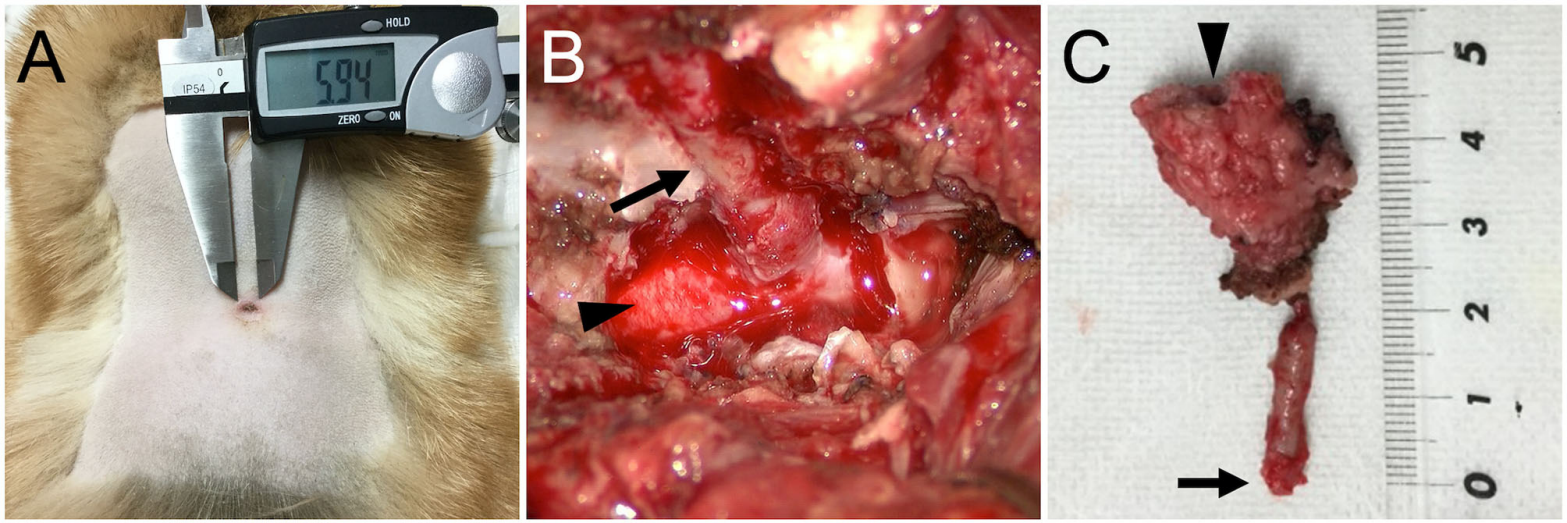
**(A)** Pre-operative photograph of a fistula on the dorsum. **(B)** Intraoperative photograph of the dermoid sinus tract (arrow) that was passing through the hollow of the conjoint spinous processes of T1–T5. The tract was composed of firm connective tissue and continuous to the dura matter of the spinal cord (arrowhead). **(C)** A photograph of the surgically excised dermoid sinus. A fistula on the surface of the skin (arrowhead) and the end of the sinus tract (arrow) are indicated.

For further examination of the spinal cord and vertebral column, MRI (Achieva 3.0T, Philips, Netherlands) was performed. MRI revealed that the findings of the spinal cord on T2-weighted imaging and T1-weighted imaging were similar to those at the referring hospital (Figures 2A,B). However, the enhanced lesion on post-contrast T1-weighted imaging after intravenous administration of contrast medium (OMNISCAN INTRAVENOUS INJECTION, Daiichi Sankyo, Tokyo, Japan) appeared larger than at examination at the referring hospital (Figure 2C). Transverse T2-weighted imaging revealed a sinus tract in the fused spinous processes between T1 and T5 that connected the spinal cord with the surface of the skin (Figure 2D). Based on the imaging finding that the sinus tract was connected to the dura mater, it was thought to be type IV dermoid sinus. Cisternal cerebrospinal fluid (CSF) analysis showed elevated total protein of 93.7 mg/dL (reference range <25 mg/dL) and normal glucose of 93 mg/dL (reference range 34–140.9 mg/dL). The differential cell count was 70% lymphocytes, 20% neutrophils, 7% monocytes, and 3% eosinophils. Bacterial culture of CSF was negative. At this stage, dermoid sinus type IV with concurrent meningitis and myelitis was suspected. A fistula on the dorsum was found after careful inspection of the skin (Figure 3A).

**Figure 4:**
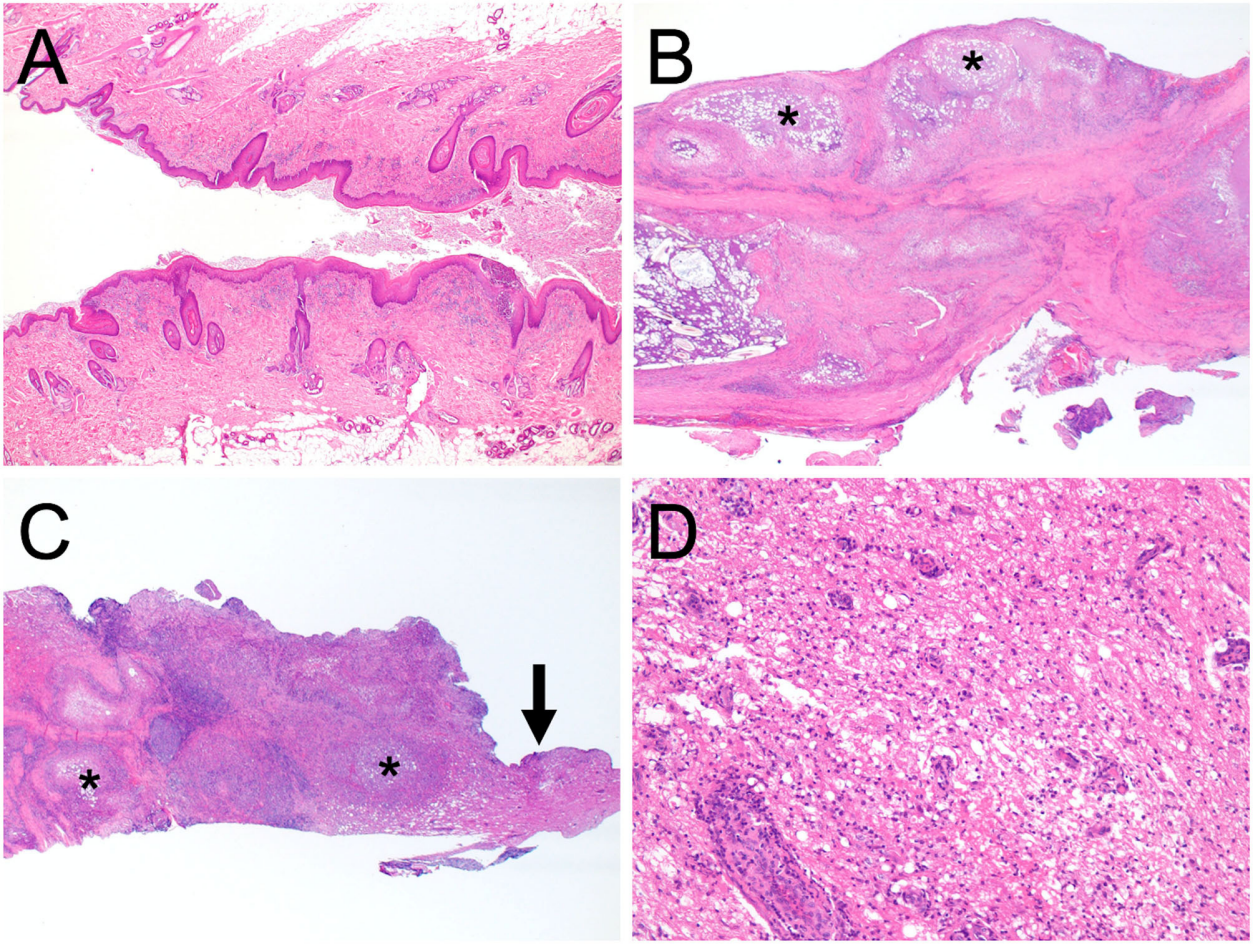
Histopathological microphotographs of the excised dermoid sinus. The resected tissues contained the orifice of the skin **(A)**, granulomas within the subcutaneous tissues **(B)**, and the intradural part of the dermoid sinus **(C)** ( ×2 magnification). Squamous epithelial cells formed follicle-like cystic structures containing sebaceous glands and sweat glands. There were connective tissues around squamous epithelial cells with infiltration of lymphocytes, plasma cells, and mast cells. Keratinous materials, clumps of bacteria, and neutrophils were present in the dermoid sinus tract. Neutrophils, lymphocytes, and macrophages invaded the intradural tissues and formed granulomas around the keratinization with hyperplasia of collagen fibers. Granulomatous inflammation is indicated (asterisk). **(D)** Corresponds with the magnified area of the intradural tissue (arrow) in **(C)**, ×200 magnification. Perivascular cuffing, edema, degeneration, and diffuse hypercellularity were observed in the excised tissue containing the spinal white matter. All sections were stained with haematoxylin and eosin.

Surgical removal of the dermoid sinus was performed on the following day. After premedication with atropine sulfate (5 μg/kg, NIPRO ES Pharma, Osaka, Japan), midazolam (0.2 mg/kg, Dormicum, Maruishi Seiyaku, Osaka, Japan), and ketamine hydrochloride (2 mg/kg, KETALAR, Daiichi-Sankyo, Tokyo, Japan), anesthesia was induced with propofol (PropoFlo 28, Zoetis Japan, Tokyo, Japan), and maintained with sevoflurane (1.7–2.1%, SevoFlo, Zoetis Japan, Tokyo) in oxygen. Fentanyl citrate (0.01–0.02 mg/kg/h, Fentanyl Injection, Janssen Pharmaceutical KK, Tokyo, Japan) and ketamine hydrochloride (0.4–0.8 mg/kg/h, KETALAR, Daiichi-Sankyo, Tokyo, Japan) were administered by continuous infusion for intraoperative analgesia. Perioperative antibiotic therapy consisted of cefazolin (20 mg/kg, Cefamezin α, LTL Pharma, Tokyo, Japan). An elliptical incision of the skin was made around the fistula. The right side of the fused spinous processes was exposed, and the muscles were expanded outward. After cutting the spinous process where the sinus tract was present with a 3-mm diamond burr, hemilaminectomy was performed to the fused T1–T5, and the sinus tract covered by the connective tissue was exposed (Figure 3B). The purulent material that drained after cutting was examined with a bacterial culture test. As the sinus tract was continuous to the dura mater, the sinus was excised after making an incision in the dura mater on the circumference of the sinus. Histological examination was performed on the excised lesion (Figure 3C). Granulomatous tissues were detached from the spinal cord parenchyma; however, most of them were firmly adhered to the spinal cord parenchyma. After irrigating the operative field with physiological saline, the dural defect was covered with subcutaneous fat tissue and fibrin glue (BeriplastP Combi-Set Tissue adhesion, CSL Behring K.K., Tokyo, Japan). The muscles, subcutaneous tissue, and skin were closed in a routine fashion. Spinal pain resolved the day after surgery. Three days postoperatively, the dog had good general condition and appetite, no signs of spinal pain were observed, and had voluntary movement in both hindlimbs. The dog was discharged 3 days after surgery. A bacterial culture test was positive for *Staphylococcus* sp. Oral administration of cefalexin (20 mg/kg q12, cefaclear, Kyoritsu Seiyaku, Tokyo, Japan) was continued for 2 months. Histopathological examination after Hematoxylin-eosin stain revealed dermoid sinus with granulomatous meningomyelitis (Figure 4). Histologically, squamous epithelial cells formed follicle-like cystic structures containing sebaceous glands and sweat glands. There were connective tissues around squamous epithelial cells with infiltration of lymphocytes, plasma cells, and mast cells. Keratinous materials, clumps of bacteria, and neutrophils were present in the dermoid sinus tract. Neutrophils, lymphocytes and macrophages invaded the intradural tissues and formed granulomas around the keratinization with hyperplasia of collagen fibers.

Follow-up examination was conducted 2 months after surgery. No signs of spinal pain were observed, and the dog was in a good condition. Although the hindlimbs were staggered, the dog was ambulatory and had voluntary urination. Cisternal CSF analysis showed a total cell count of 2.5 cells/μL, total protein of 18.4 mg/dL, and glucose of 74 mg/dL. CSF culture was negative for bacteria. On MRI, the spinal cord at the level of the T5–T6 vertebrae presented high signal intensity on sagittal T2-weighted imaging, but this region was not enhanced on T1-weighted imaging with intravenous administration of a contrast medium. The latest follow-up was performed 4 months after surgery. The dog's general condition was good, and it was ambulatory and had voluntary urination. No signs of spinal pain were observed, but proprioceptive deficits remained in both hindlimbs. Cisternal CSF analysis showed: total cell count of 0 cells/μL, total protein of 16.8 mg/dL, and glucose of 71 mg/dl. Bacterial culture test of CSF was negative. On MRI, the spinal cord at the level of the T5–T6 vertebrae presented a high signal intensity on sagittal T2-weighted imaging, as in the previous follow-up, and no contrast enhancement effect was observed on the T1-weighted imaging.

## Discussion

Dermoid sinus is a congenital malformation resulting from failure of separation of the neural tube from the skin ectoderm during embryonic development (1, 2). Dermoid sinus is associated with vertebral malformations including spina bifida, block vertebrae, or hemivertebrae in dogs and cats (9, 10, 13–20). The present dog had lordosis of the thoracic vertebrae, fused spinous processes of T1–T5, and dermoid sinus. The tract of the dermoid sinus passed through a bone tunnel of the fused spinous processes. In a previous study of a cat with dermoid sinus, the tract of the dermoid sinus was present in the partially fused spinous processes of T1–T2 (19); however, there were no studies of dermoid sinus associated with fused spinous processes in dogs. A block vertebra is a congenital vertebral anomaly resulting from fusion of two or more vertebrae (2). Fusion of vertebrae may be limited to vertebral bodies or involve related vertebral arches (21). A block vertebra is caused by the failure of segmentation that occurs when two adjacent somites or their associated mesenchyme do not separate properly during embryogenesis (2, 22). In addition, the lordosis found in the present case was a congenital abnormality associated with a defect of vertebral segmentation (22). The present dog had only 11 thoracic vertebrae and the ribs of T3 and T4 were abnormally thickened, suggesting the segmentation in the T3–T4 region was incomplete. Although the detailed mechanism was not clear, the dermoid sinus and vertebral malformations identified in the same region in this case were thought to be related.

In the present case, information about family history was unknown and genetic testing was not performed, so the pathogenesis of the dermoid sinus was unknown. In Rhodesian ridgeback, the dermoid sinus is suspected to be an inherited neural tube defect (4, 23). Moreover, a dominant mutation in the fibroblast growth factor (FGF) genes that form the dorsal hair ridge has been identified and considered to be a predisposing factor of dermoid sinus in this breed (4, 23). The FGF genes play an important role during development and their dysregulation causes the ridge and dermoid sinus (4). However, a recent study concluded that the 133-kb duplication involving three FGF genes was not identical with the hypothesized locus for dermoid sinus (5). Dermoid sinus has been reported in several other breeds such as Yorkshire terrier, American cocker spaniel, English springer spaniel, Chow chow, Golden retriever, Siberian husky, French bulldog, Dachshund, Chinese crested dog, Swedish vallhund, Victorian bulldog, and Cane corso (8–11, 13–17, 20, 24–26). The mode of inheritance in these dogs other than Rhodesian ridgeback has not been clarified, and dermoid sinus is considered to be sporadic. There are no reports of dermoid sinus in Shiba Inu.

Different imaging techniques such as myelography, fistulography, MRI and CT can be useful for diagnosis of dermoid sinus (3, 18, 19). Myelography or fistulography have been used to evaluate the extent of the sinus tract, but they have a risk of causing myelitis (18). In some reports, MRI was useful in diagnosing dermoid sinus and evaluating the extent of the sinus tract, while other reports described that the extent of the sinus tract could not be determined based on the MR images (9, 14, 19). A report on the use of CT in the diagnosis of dermoid sinus in two dogs described that single-phase contrast CT was useful to accurately determine the extent of the sinuses (18). In the present case, the combination of MRI and CT allowed for the diagnosis and determination of the extent of dermoid sinus.

Dermoid sinus has a good prognosis with complete surgical excision, whereas the prognosis in cases with neurological signs is guarded (12). However, there is little prognostic information about cases with spinal malformations and severe inflammatory granuloma in spinal cord secondary to infection. In Type IV dermoid sinus, neurological signs are caused by the communication between the sinus and the dura mater, which causes meningomyelitis or myelitis secondary to infection (9, 11). In this case, preoperative MR images showed enhanced lesion in the spinal cord, suggesting inflammatory granuloma, which was confirmed by postoperative histopathological examination. The neurological signs were thought to be due to severe inflammation of the spinal cord, which was caused by bacterial infection and infiltration of granuloma into the spinal cord parenchyma. In a report describing bacterial meningoencephalomyelitis in dogs, CBC and serum chemistry were abnormal in 57 and 71%, respectively (27). The study also found that only 13% of dogs diagnosed with bacterial meningoencephalomyelitis based on postmortem examination were positive by CSF culture (27). In the present dog, CBC and blood biochemical examination were unremarkable, despite the presence of severe meningomyelitis caused by bacterial infection. In addition, preoperative bacterial culture of CSF was negative. These findings may be due to the antibiotic treatment being prescribed by the referral hospital. Thus, even if CBC and blood biochemical examination are normal and the preoperative bacterial culture of CSF is negative, the possibility of bacterial infection caused by dermoid sinus should not be ruled out. In the present case, CSF analysis showed increased total cell count and total protein, which suggested an inflammatory lesion in the spinal cord. As the inflammation becomes chronic, the granuloma becomes more severe, and it could be difficult to completely remove from the spinal cord parenchyma by surgery. Thus, if dermoid sinus that reaches the dura mater (type IV or VI) is suspected, CSF analysis should be performed to evaluate the presence of spinal cord inflammation.

The sinus tract passed through the tunnel of fused spinous processes in the present case, so the right side of the spinous processes was removed to expose and resect the sinus tract. Postoperative antibiotic treatment was performed for 2 months, and at the time of follow-up 4 months after surgery, the dog was ambulatory and had voluntary urination. Although long-term follow-up is required, based on the progress of this case, it is considered that surgical resection is effective even in dogs with dermoid sinus with severe granuloma secondary to bacterial infection and spinal malformations.

## Data Availability Statement

The raw data supporting the conclusions of this article will be made available by the authors, without undue reservation.

## Ethics Statement

Written informed consent was obtained from the owners for the participation of their animals in this study.

## Author Contributions

YN, SK, TI, KT, SM, and HK helped with the diagnosis of this case and participated in clinical case management. JC performed the pathological studies and interpretation of the results. KT and HK participated in the writing and editing the manuscript. All authors contributed to the article and approved the submitted version.

## Conflict of Interest

The authors declare that the research was conducted in the absence of any commercial or financial relationships that could be construed as a potential conflict of interest.

## Publisher's Note

All claims expressed in this article are solely those of the authors and do not necessarily represent those of their affiliated organizations, or those of the publisher, the editors and the reviewers. Any product that may be evaluated in this article, or claim that may be made by its manufacturer, is not guaranteed or endorsed by the publisher.
